# Intact anti‐LPS IgY is found in the blood after intragastric administration in mice

**DOI:** 10.1002/2211-5463.12571

**Published:** 2019-01-30

**Authors:** Xin Zhou, Pei Wang, Yajie Chen, Si‐yuan Ma

**Affiliations:** ^1^ Institute of Burn Research State Key Laboratory of Trauma, Burns and Combined Injury Third Military Medical University (Army Medical University) Chongqing China

**Keywords:** absorption, egg yolk antibody, infection, lipopolysaccharide, mammalian gut

## Abstract

Severe burn injury and cirrhosis often cause the translocation of bacterial endotoxins into blood, leading to systemic damage and even death. Our previous studies have shown that anti‐lipopolysaccharide egg yolk antibody (anti‐LPS IgY) can neutralize bacterial endotoxins *in vitro* and *in vivo* effectively, thereby reducing endotoxin damage. Whether anti‐LPS IgY can be absorbed into the blood through the intestinal barrier and neutralize endotoxins in circulation remains unclear. In this study, we used *in vivo* small animal imaging techniques, protein purification, molecular biology, and mass spectrometry to show that intragastrically administered anti‐LPS IgY is detected in the blood of mice as an intact molecule and has the capacity to bind to LPS. Immunohistochemical analysis confirmed that anti‐LPS IgY is associated with the intestinal mucosa of mice. However, the route of absorption of this large protein molecule was not determined. This study suggests that anti‐LPS IgY can be absorbed into the circulation, with the same molecular mass as purified anti‐LPS IgY as a macromolecular protein, suggesting a new strategy for the prevention of damage caused by endotoxins.

**Abbreviations**
BHC: heavy chain of botulinum neurotoxin
CCK: cholecystokinin
FcRn: neonatal Fc receptor
IgY: immunoglobulin Y or egg yolk antibody
LPS: lipopolysaccharide
MALDI‐TOF MS: matrix assisted laser desorption ionization time of flight mass spectrometry
WB: western blot

Lipopolysaccharide (LPS), also referred to as endotoxins, in the outer membrane of Gram‐negative bacteria comprise O‐specific side chains, core polysaccharides and lipid A, and exhibit a wide range of biological activities with important roles in septic shock, diffuse intravascular coagulation and multi‐organ failure.

At present, although the treatment of LPS injuries has advanced, sepsis and other LPS‐related diseases continue to be one of the major causes of death in patients in intensive care units worldwide, presenting an increasing trend in recent years 1. The study of anti‐endotoxin drugs is one of the most active fields in medicine and pharmacology. There are numerous inter‐relationships and complex inflammatory pathways involving LPS. None of the antagonists of a single molecule in the pathway, such as cluster of differentiation 14, lipopolysaccharide‐binding protein, and toll‐like receptors 4, could achieve satisfactory effects. Additionally 2, tumor necrosis factor‐α and other important molecules perform physiological functions in the body. The use of large doses of antibody to block the functions of these molecules leads to notable side effects.

Egg yolk antibody (IgY) is an immunoglobulin from poultry with a molecular mass of ~ 186 kDa. Specific IgY can be harvested from hens immunized with antigen or purified from eggs by many methods, such as the water dilution method of gel filtration chromatography. IgY has specific antigen‐binding sites and Fc segments that play roles in neutralizing specific immunogens. Our previous studies have shown that anti‐LPS IgY could improve the symptoms caused by LPS 3. As we know, the absorption of orally administered anti‐LPS IgY into the blood through the intestinal barrier to effectively neutralize endotoxins in circulation is key to preventing and treating endotoxin damage. However, it is not clear whether anti‐LPS IgY can be absorbed into the circulation from the intestinal tracts of mammals. Confirmation that anti‐LPS IgY can be effectively absorbed into mammalian circulation will be helpful in developing new oral drugs for preventing endotoxin damage, particularly intestinal endotoxin damage, with good compliance.

Studies have shown that anti‐cholecystokinin (CCK)‐specific IgY orally administered to Sprague–Dawley rats could be absorbed into the blood from the duodenum and promoted the growth of animals, partly by eliminating the satiety effect of endogenous CCK 4. Another study showed that the oral administration of CCK yolk antibody could partially neutralize CCK in circulation 5. The United States Department of Agriculture reported that the mastitis symptoms of dairy cows were obviously reduced after rearing with IgY anti‐cow mastitis virus for 3 weeks at a dose of 200 mg·kg^−1^·day^−1^ 6. The intragastric administration of anti‐heavy chain of botulinum neurotoxin (BHC) IgY can prevent a fatal attack of tetanus toxin, and the intraperitoneal injection of anti‐BHC IgY could play a protective role in mice 7. Our previous studies have also shown that the oral administration of anti‐LPS IgY could reduce the mortality rate and levels of inflammatory factors in the blood of mice attacked by endotoxin 3, 8. Lu *et al*. 9 found that the continuous feeding of white spot syndrome virus yolk antibodies for 10 days could significantly reduce the death caused by that virus in shrimp. Based on these results, we concluded that IgY, which targets certain antigens, could exert systemic biological effects against the corresponding antigens through feeding. Thus, we supposed that IgY could have a role in cows, rats and mice by absorption from the intestine into the circulation, subsequently reaching target tissues or organs to neutralize the corresponding antigens. Thus, although IgY is a macromolecular protein, this molecule can bind to the intestinal tract of mammals and be absorbed into the blood to exert biological effects.

IgY has excellent stability in heat and acid. After intragastric administration, it can reach the small intestine of mammals as an intact molecule with biological activity 10, 11. It could be used as a representative for studying the oral absorption of proteins from the intestine. The United States Food and Drug Administration has determined IgY to be a generally accepted safe substance for oral administration 12, providing a basic guarantee and showing potential for the large‐scale oral administration of IgY in the prevention and treatment of related diseases in the future. In this study, live animal imaging, protein purification, molecular biology and mass spectrometry (MS) were used to elucidate whether IgY could be absorbed into the intestinal tracts of mice as a macromolecular protein.

## Materials and methods

### Materials

#### Anti‐LPS IgY preparation

We produced antibodies against LPS (anti‐LPS IgY) from egg yolk in our previous work 13. Briefly, LPS from *Escherichia coli* (O111:B4) mixed with Freund's adjuvant was used as the immunogen to immunize Roman hens. Immunized eggs were collected, and IgY was purified using a water solution, salt precipitation and gel chromatography.

#### Purification of FITC‐labeled IgY

Anti‐LPS IgY was prepared at a concentration of 20 mg·mL^−1^ in 0.9% NaCl solution and carbonate buffer. Anti‐LPS IgY and FITC were mixed at an IgY: FITC ratio of 100 : 1. The mixture was shaken at 4 °C for 12 h and centrifuged at 1048 ***g*** for 20 min. The supernatant was placed in a carbonate buffer, pH 8.0, overnight at 4 °C and subsequently centrifuged again at 1000 ***g*** for 5 min. The supernatant was placed in an ÄKTAexplorer Primer protein purification instrument (GE Healthcare, New York, NY, USA). FITC–IgY was collected using a G25 chromatography column in outflows of P1 peak and quantified at *A*_280nm_.

#### Intragastric administration of FITC–IgY

Adult Nude mice (male or female) were provided by the Laboratory Animal Center, Army Medical University, and housed in cages in a standard animal room. The experimental procedure was approved by the Ethical Committee of Southwest Hospital, Army Medical University. The mice were conventionally fed for 3 days and did not receive food or drink for 24 h prior to the experiment. The mice were divided into the following two groups: the normal control group (intragastric administration of normal saline at 0.5 mL per mouse) and the FITC–IgY intragastric administration group (intragastric administration of FITC–IgY at 0.5 mg per mouse). In the normal control group and the experimental group, the six phase points were set up for 30 min and 1, 2, 8, 12 and 24 h, and two normal mice and two experimental mice were used at each time point. Normal control mice were administrated with 0.5 mL normal saline before the experiment. Mice in the experimental group were intragastrically administered with normal saline containing 0.5 mg IgY before the beginning of the experiment. At all later points intragastric administration was not performed, and fluorescence was observed only. With continuous water fasting, the distribution of FITC fluorescence in mice was observed by a small animal living imaging instrument at 30 min and 1, 2, 8, 12 and 24 h later under anesthesia through an intraperitoneal injection of sodium phenobarbital (60 mg·kg^−1^). The results showed that the fluorescence intensity was strongest in mice at 8 h after the intragastric administration of FITC–IgY. Therefore, the vital organs, such as the heart, liver, spleen, lungs, kidneys and intestines, were dissected and harvested according to routine surgical anatomy on the operating table from the mice anesthetized by intraperitoneal injection of sodium pentobarbital (1%, 50 mg·kg^−1^) after the intragastric administration of FITC‐labeled anti‐LPS IgY, and the fluorescence intensity was observed by a small animal living imaging instrument to determine the FITC–IgY distribution. The study was repeated in three replicates.

#### ELISA

ELISAs were conducted according to the manufacturer's instructions (BOSTER Biological Technology, WuHan, China). Briefly, 96‐well plates were coated with LPS (100 μg·mL^−1^) carbonate buffer for 48 h at 4 °C. The blood harvested from the mice after the intragastric administration of FITC‐labeled anti‐LPS IgY was diluted 1 : 400, 1 : 1600, 1 : 3200, 1 : 6400, 1 : 12 800 and 1 : 25 600 using antibody dilution. Then, the diluted blood was added to the pre‐coated 96‐well plates at 200 μL per well. Two double holes per group and blank (antibody dilution only), negative (non‐immune IgY only) and positive (blood harvested from mice of intraperitoneal injection of anti‐LPS IgY) control groups were set up. Following incubation for 1 h at 37 °C and subsequent washing, horseradish peroxidase‐labeled sheep anti‐chicken secondary antibody at a dilution of 1 : 5000 was added to the plates at 200 μL per well and incubated for another 1 h at 37 °C. After washing three times, 3,3′,5,5′‐tetramethylbenzidine solution was added to each well and incubated in the dark for 30 min. Termination liquid (2 m sulfuric acid) was added to the wells at 100 μL per well to stop the reaction. The signal was detected on a microplate reader at 450 nm.

#### Western blot analyses

To increase the content of target protein in the blood for detection, the intragastric administration of anti‐LPS IgY was repeated every 2 h for up to four times until 8 h after the first administration. In the intraperitoneal injection group, a single injection of anti‐LPS IgY was administered to mice. Eight hours later, the mice were sacrificed, and blood samples were collected. To remove the interference of IgG in serum, the ProteoExtract Albumin/IgG Removal Kit (Merck Millipore, Bedford, MA, USA) was used. Finally, SDS/PAGE and western blot (WB) were carried out to detect the molecular mass of target proteins and identify their species. SDS/PAGE was conducted under reducing conditions with a 10% gel on an electrophoresis apparatus (Bio‐Rad, Hercules, CA, USA). After electrophoresis, the gel was stained with Coomassie brilliant blue G‐250 and destained with deionized water. The gel was analyzed using image analysis software with a Universal Hoop II Imaging Gel analyzer (Bio‐Rad). Finally, the protein band at 186 kDa was selected for western blotting assay using sheep anti‐chicken secondary antibody. A normal mice group (blank control), intragastric administration of anti‐LPS IgY group and intraperitoneal injection of anti‐LPS IgY group (positive control) were set up.

#### MALDI‐TOF MS analysis

The mice were divided into the following two groups: the intragastric administration group (intragastric administration of 0.5 mg anti‐LPS IgY) and the intraperitoneal injection group (intraperitoneal injection of 0.5 mg anti‐LPS IgY). After 8 h, the mice were sacrificed, and the plasma was harvested. Protein bands near 180 kDa were isolated by SDS/PAGE electrophoresis according to Ma and Zhang 13. After the protein was digested by trypsin, the peptides were analyzed by matrix assisted laser desorption ionization time of flight (MALDI‐TOF) MS (HPLC‐CHIP‐MS/MS ION TRAP 6330; Agilent, Santa Clara, CA, USA). Purified anti‐LPS IgY was used as a standard control (the anti‐LPS IgY preparation described earlier). The plasma from mice injected intraperitoneally with anti‐LPS was used as a positive control. The data were analyzed using mill proteomics software (RevA.03.03.060; Agilent).

#### Immunohistochemistry and immunofluorescence

The intestines of mice were dissected and harvested after intragastric administration of FITC‐labeled anti‐LPS IgY 8 h later only. The intestinal tissue with the strongest fluorescence intensity was selected under the small animal live imager instrument, and the slices were prepared. FITC‐labeled anti‐LPS IgY was assessed by immunohistochemical and immunofluorescence staining 14.

### Statistical analysis

We carried out the experiments of the immunohistochemical, immunofluorescence and intragastric administration of FITC–IgY in three replicates. In ELISA, we set the each kind of sample for three replicates too. spss 16.0 (SPSS Inc., Chicago, IL, USA) was used for the statistical analysis. ANOVA was used to analyze the results of ELISA.

## Results

### Purification of FITC‐labeled IgY

**Figure 1:**
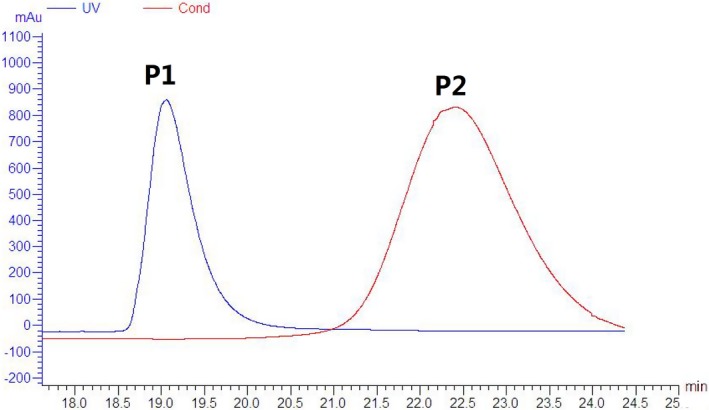
Purification of FITC‐labeled IgY by G25‐filled gel chromatography column. P1: FITC‐anti‐LPS IgY; P2: free FITC. Cond, Conductivity curve; UV: UV, scanning curve.

The FITC–IgY and free FITC could be well separated with a G25‐filled gel chromatography column. The peak at P1 represents the FITC–IgY protein elution, and the peak at P2 is a free FITC peak outflow (Fig. 1). The target protein, FITC‐labeled anti‐LPS IgY, could be obtained by collecting the P1 peak effluent (Fig. 1).

### Distribution of FITC‐labeled anti‐LPS IgY

**Figure 2:**
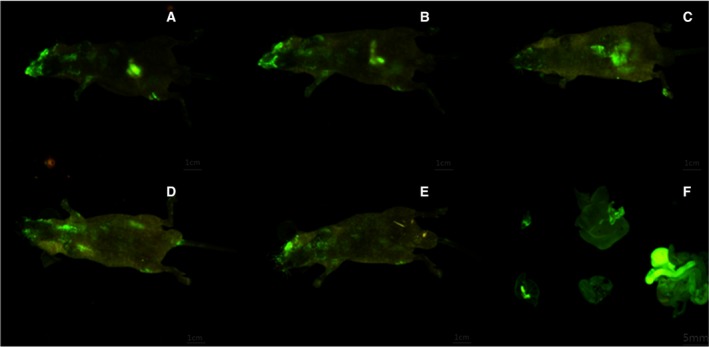
Distribution of FITC‐labeled anti‐LPS IgY after intragastric administration to mice. At 30 min after the oral administration of FITC‐IgY, green fluorescence was observed in the abdominal and thoracic cavities of mice. Clear green fluorescence was accumulated in mice from 30 min to 8 h after intragastric administration. After 8 h, the fluorescence gradually began to weaken and disperse and almost disappeared at 24 h later. At 8 h after intragastric administration, the main organs, such as the intestine, liver, lungs and kidneys, all showed fluorescence distribution. However, the fluorescence intensity in different viscera markedly differed. (A–E) 30 min (A), 1 h (B), 2 h (C), 8 h (D), and 24 h (E) after intragastric administration. (F) The main organs 8 h after intragastric administration (heart, liver, spleen, lungs, kidneys and intestine in the order of top to bottom and left to right).

At 30 min after the oral administration of FITC–IgY, the distribution of green fluorescence could be observed in the abdominal and thoracic cavities of mice (Fig. 2). The normal mice showed no fluorescence (data not shown). Clear green fluorescence was accumulated in mice from 30 min to 8 h after intragastric administration. After 8 h, the fluorescence gradually began to weaken and disperse and almost disappeared after 24 h. We also found that at 8 h after intragastric administration, the main organs, such as the intestine, liver, lungs and kidneys, all showed fluorescence distribution. However, the fluorescence intensity in different organs markedly differed. At 8 h after intragastric administration, large amounts of IgY had not yet been emptied. However, a portion of the proteins had been absorbed into the blood across the intestinal barrier and distributed in the liver, kidneys and other organs (Fig. 2).

### IgY binding to the mouse intestine

**Figure 3:**
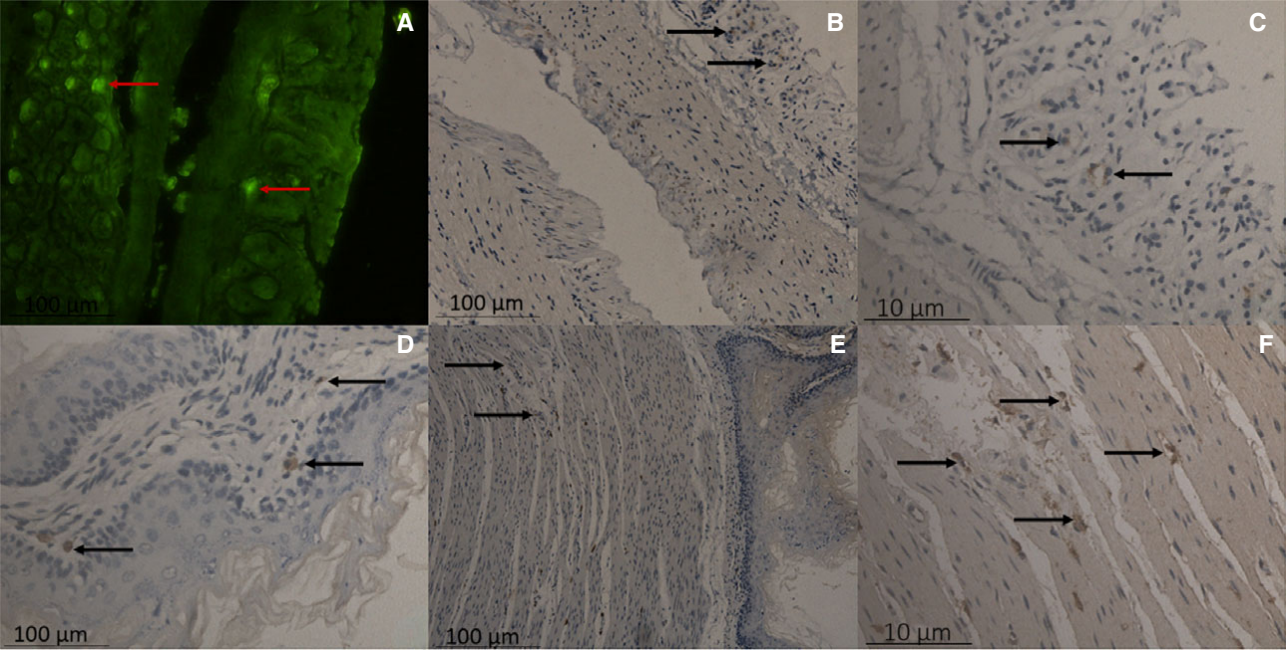
IgY binding to intestinal mucosal tissue, as shown by immunofluorescence. Fluorescent protein molecules were immunohistochemically detected with sheep anti‐chicken secondary antibodies (A) and shown by immunohistochemistry (B–F). Scale bar: 100 μm (A,B,D,E), and 10 μm (C,F).

The intestinal tracts of the mice were dissected, the small intestines with the strongest fluorescence were selected for sectioning, and the results showed a significant amount of fluorescence distributed in the intestinal mucosa at 8 h after the intragastric administration of FITC–IgY. These fluorescent protein molecules could be immunohistochemically detected with sheep anti‐chicken secondary antibody, confirming that the fluorescent protein molecules were derived from the intragastrically administered FITC‐labeled anti‐LPS IgY (Fig. 3).

### Binding of blood to LPS

**Figure 4:**
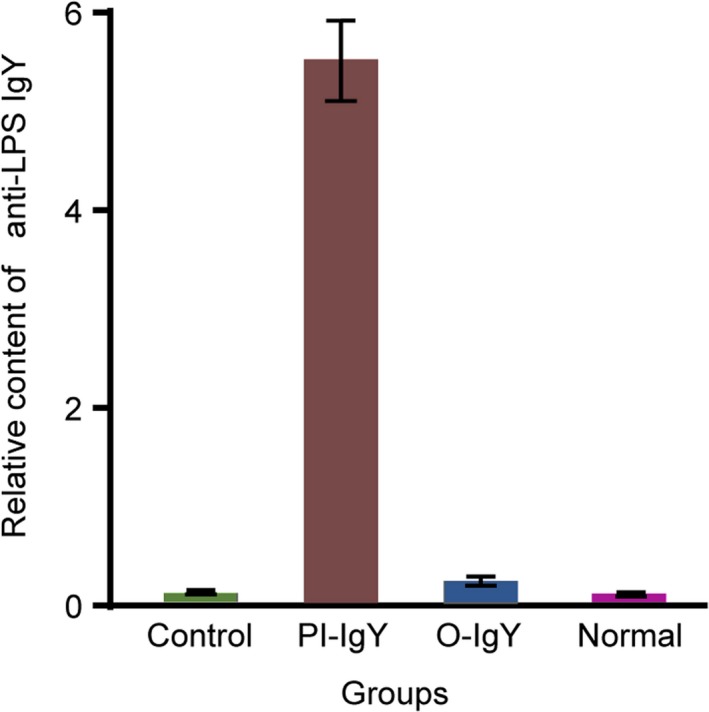
Antibody titre of anti‐LPS IgY (absorbance value) in blood harvested from mice. After the administration of anti‐LPS IgY, the plasma of mice had the ability to bind to LPS, as tested by ELISA. The absorbance of the blood from the oral administration group (0.27 ± 0.12) was significantly higher than that of the blood from the normal group (0.05 ± 0.00; *P* = 0.000) by ANOVA. The ability to bind to LPS was greater in the blood of mice intraperitoneally injected with anti‐LPS IgY absorbance value, 5.77 ± 0.39; the absorbance value of blank control was 0.05 ± 0.01). Control, PBS solution; O‐IgY, mice orally administered IgY; PI‐IgY, mice administered IgY by peritoneal injection; Normal, mice without IgY administration (*n* = 2).

Plasma was acquired from mice intragastrically administered anti‐LPS and IgY, and ELISA revealed that the plasma could bind to LPS (absorbance value, 0.27 ± 0.12). The plasma of normal mice did not bind to LPS (absorbance value, 0.05 ± 0.00). The ability to bind to LPS was greater in the blood of mice intraperitoneally injected with anti‐LPS IgY (absorbance value, 5.77 ± 0.39; the absorbance value of blank control was 0.05 ± 0.01) (Fig. 4).

### Intact IgY in mouse circulation

The above experiments showed that after the intragastric administration of FITC‐labeled anti‐LPS IgY, there was protein in the blood that could bind to LPS, but we could not determine whether the protein molecules were intact IgY or active fragments of IgY digested by enzymes. Therefore, we used MS to determine the molecular mass of the corresponding proteins in the blood.

**Figure 5:**
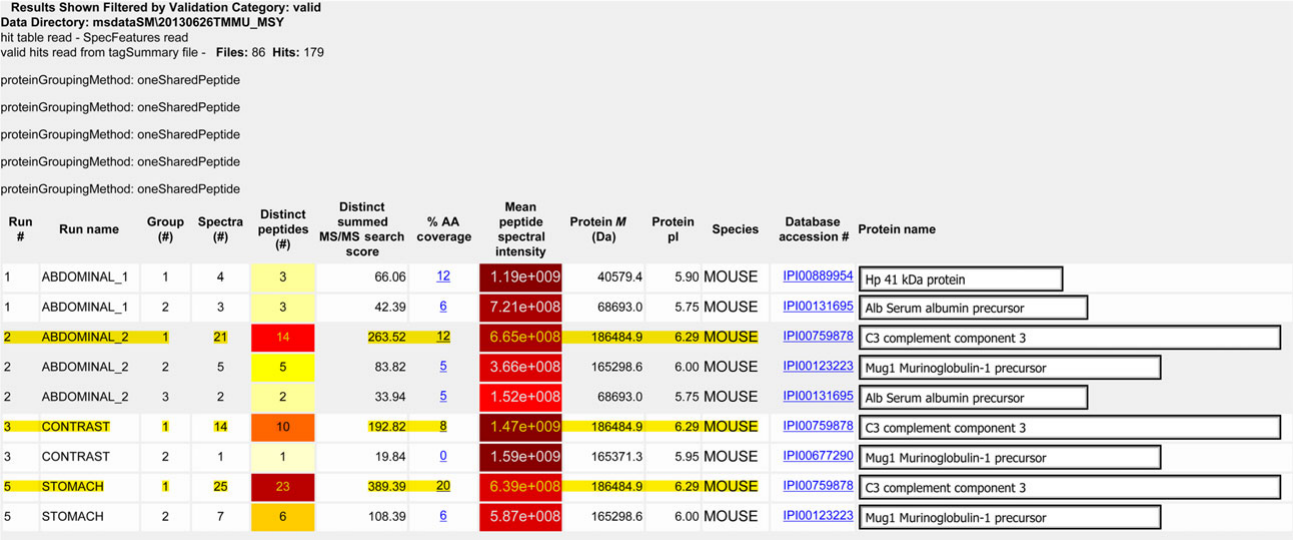
HPLC‐MS/MS of proteins harvested from mouse blood. Protein with a molecular mass of 186.4 kDa could be detected in the blood of mice after the intragastric administration and intraperitoneal injection of anti‐LPS IgY, a size identical to that of the purified standard anti‐LPS IgY. Stomach, mice orally administered IgY; Abdominal, mice administered IgY by peritoneal injection; Contrast, pure anti‐LPS IgY.

The results showed protein molecules with a molecular mass of 186.4 kDa in the blood of mice after the intragastric administration and intraperitoneal injection of anti‐LPS IgY, a size identical to that of the purified standard anti‐LPS IgY (Fig. 5). Thus, after the intragastric administration of anti‐LPS IgY, the intact IgY molecule was absorbed into the blood across the mouse intestinal mucosal barrier.

### Protein binding to LPS in the blood of mice

**Figure 6:**
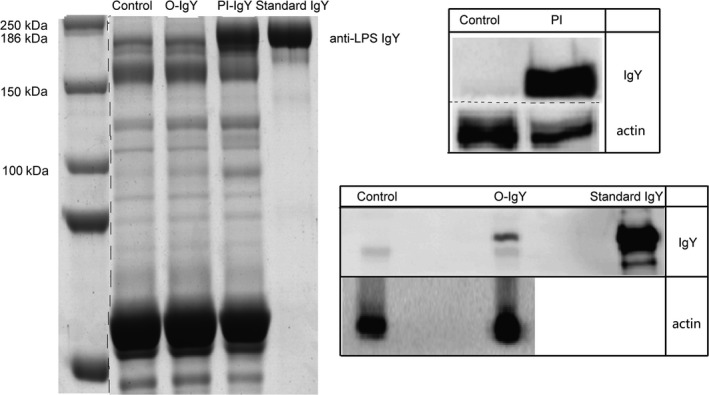
SDS/PAGE (left) and WB (right) of blood harvested from mice. After the intragastric administration of anti‐LPS and IgY, particularly after the intraperitoneal injection of anti‐LPS IgY, proteins with a molecular mass of 186.4 kDa could be detected in the blood of mice by sheep anti‐chicken secondary antibodies. Control, mice without the administration of IgY; O‐IgY, mice orally administered IgY; PI‐IgY, mice administered IgY by peritoneal injection; Standard IgY, pure anti‐LPS IgY. All lanes come from the same gel, but dashed lines indicate where lanes have been spliced together.

After the intragastric administration of anti‐LPS and IgY, particularly after the intraperitoneal injection of anti‐LPS IgY, protein with a molecular mass of 186.4 kDa could be detected in the blood of mice, and it combined with sheep anti‐chicken monoclonal antibody to show colored protein bands. These protein bands could not be detected in normal mouse plasma (Fig. 6).

## Discussion

The oral administration of drugs has several advantages, including patient compliance, ease of administration and reasonably low cost of production. The oral delivery of peptides and proteins is now a topic of intense research. Advancements in the biopharmaceutical industry have resulted in the development of several new protein‐ and peptide‐based therapeutics. Oral administration is most preferred because of patient compliance and acceptability 15. However, the intestinal barrier restricts the absorption of most protein drugs administered orally 16, 17.

Immunoglobulin Y, as a macromolecular protein derived from poultry, has the advantages of high yield, no blood collection, simple purification and low cost 18; however, humanized protein is expensive, complex in preparation and low yielding, and cannot meet the large demand as an oral drug. Therefore, the development of yolk antibodies with specific effects against bacteria has significant application prospects, especially for less‐developed areas. The low cost of access will greatly reduce the cost of treatment and benefit more patients. However, whether these antibodies are absorbed as macromolecular proteins into the circulation by the mammalian intestine has become an important factor restricting the effectiveness and wider application of IgY.

Our study found that after the intragastric administration of FITC–IgY, there was fluorescence distribution in many organs of the mice. Although the fluorescently labeled protein may enter the blood through the intestine and be distributed throughout the body, we cannot completely exclude the possibility that FITC entered into the blood after intragastric administration. The blood of the mice showed the ability to specifically bind LPS only after the FITC–IgY entered the circulation from the gut. Thus, we collected blood samples from mice intragastrically administered FITC–IgY at 8 h later and tested the binding activity of IgY to LPS by ELISA. Additionally, intraperitoneal injection of FITC–IgY was used as a positive control. After the intragastric administration of FITC–IgY, the blood of mice showed the ability to specifically bind LPS, although with lower affinity than that of the intraperitoneal injection groups. These results indicated that the biologically active form of intact IgY or its fragments, which could bind LPS, was absorbed into the blood after the intragastric administration of FITC–IgY.

To further confirm these results, we determined whether the protein molecules with the ability to bind to LPS that appeared in the circulation of mice were intact IgY or the fragments of IgY digested by enzymes. We determined the molecular mass of the proteins by MS. The results showed that the proteins in the blood of the mice were identical to those of the purified anti‐LPS IgY, with the molecular mass of 186 484.9 Da. Thus, the protein present in the blood of the mice orally administered IgY was intact IgY, not fragments resulting from enzymatic hydrolysis.

Taken together, these results suggested that after the intragastric administration of IgY, the proteins present in the blood of mice were identical to those of standard IgY with the same molecular mass and specific LPS‐binding activity. However, whether the protein was derived from poultry or from the mouse itself with the same molecular mass as IgY is still unknown. WB using the sheep anti‐chicken‐specific antibody as the secondary antibody demonstrated that the protein present in the blood of mice was IgY derived from poultry, not from mice. After the intragastric administration of IgY, the protein bands in the blood showed the same molecular mass as that of the standard anti‐LPS IgY and could be expressed by sheep against chicken secondary antibody. This result was also consistent with the presence of protein bands in the blood after the intraperitoneal injection of IgY.

In summary, our results showed that after oral administration of anti‐LPS IgY, there were proteins present in the blood with the molecular mass of 186 484.9 Da, which was identical to that of the purified standard IgY, with the ability to bind LPS, which could be labeled by the sheep anti‐chicken monoclonal antibody. Thus, these proteins were intact anti‐LPS IgY, and we propose that after intragastric administration, anti‐LPS IgY could be absorbed into the blood from the mouse gut as an intact molecule.

Previous studies have suggested that there may be other methods of absorption after the oral administration of IgY. That is, intact IgY molecules were digested by enzymes into small biologically active fragments and then absorbed into the blood from the intestinal tracts of mice. This study did not rule out this possibility. Therefore, we did not measure the absorption rate and bioavailability of IgY orally administered in this study. We only conducted qualitative studies to determine whether this molecule could be absorbed in the guts of mice, and not quantitative studies to confirm the absorption rate and bioavailability. Indeed, the use of various intestinal absorption enhancers could significantly improve the oral absorption efficiency.

The traditional theory holds that macromolecular proteins entering the mammalian gut are digested by enzymes and then absorbed into the blood as amino acids or peptides from the mammalian intestine, not in the form of an intact protein molecule with biological activity. However, other studies have also found that certain proteins, such as natto kinase and earthworm kinase, could be absorbed into the blood through the intestinal epithelium of mammals as intact molecules. These proteins were absorbed into the circulation from mouse gut as intact molecules 7, 19, 20. Our study confirmed these findings, although the detailed processes remain unclear.

We further examined intestinal biopsy sections and performed immunohistochemistry. The results showed that after the intragastric administration of FITC‐labeled anti‐LPS IgY, IgY was absorbed from the lumen of intestinal mucosa to the submucosal capillary cavity. However, macromolecular protein transport is not achieved through simple diffusion or leakage, and the most likely route is carrier transport‐mediated endocytosis. A recent study showed that IgG could be absorbed from the gut into the blood by receptor‐mediated endocytosis through a neonatal Fc receptor (FcRn), a new protein transporter found on the neonatal intestinal epithelial surface 11, 12, 21. However, studies have shown that due to the structural differences in proteins, the Fc fragment of IgY cannot bind to the Fc receptor on mammalian cell membranes. Thus, IgY does not undergo endocytosis and transport into blood by FcRn 22. Other studies have indicated that surface modifications of proteins by hydrophobization could improve transport. IgY also has hydrophobic properties. Thus, although anti‐LPS IgY is a macromolecular protein with a molecular mass of 186.49 kDa, absorption into the blood through the mammalian intestine by transcytosis via a similar or unknown receptor is feasible. Perhaps laser colocalization, coimmunoprecipitation, the glutathione‐*S*‐transferase fusion protein pull‐down technique, and far western blotting will be helpful to isolate the transporter or receptor and investigate how IgY binds to mammalian intestinal cells and is absorbed into the circulation from the intestine. This work is ongoing in our lab.

## Author contributions

SM conceived and designed of the study, participated in the sequence alignment and statistical analysis, and wrote the manuscript. PW carried out part of the molecular biology studies. YC performed the animal experiments. XZ participated in the design of the study and molecular biology studies, and also the animal experiments. All authors read and approved the final manuscript.

## Conflict of interest

The authors declare no conflict of interest.
